# Temporal expression of mitochondrial life cycle markers during acute and chronic overload of rat plantaris muscles

**DOI:** 10.3389/fphys.2024.1420276

**Published:** 2024-08-30

**Authors:** Jon-Philippe K. Hyatt, Emilie J. Lu, Gary E. McCall

**Affiliations:** ^1^ College of Integrative Sciences and Arts, Arizona State University, Tempe, AZ, United States; ^2^ Department of Exercise Science, University of Puget Sound, Tacoma, WA, United States

**Keywords:** biogenesis, oxidative phosphorylation, myosin heavy chain, mitophagy, autophagy, MOTS-c

## Abstract

Skeletal muscle hypertrophy is generally associated with a fast-to-slow phenotypic adaptation in both human and rodent models. Paradoxically, this phenotypic shift is not paralleled by a concomitant increase in mitochondrial content and aerobic markers that would be expected to accompany a slow muscle phenotype. To understand the temporal response of the mitochondrial life cycle (i.e., biogenesis, oxidative phosphorylation, fission/fusion, and mitophagy/autophagy) to hypertrophic stimuli, in this study, we used the functional overload (FO) model in adult female rats and examined the plantaris muscle responses at 1 and 10 weeks. As expected, the absolute plantaris muscle mass increased by ∼12 and 26% at 1 and 10 weeks following the FO procedure, respectively. Myosin heavy-chain isoform types I and IIa significantly increased by 116% and 17%, respectively, in 10-week FO plantaris muscles. Although there was a general increase in protein markers associated with mitochondrial biogenesis in acute FO muscles, this response was unexpectedly sustained under 10-week FO conditions after muscle hypertrophy begins to plateau. Furthermore, the early increase in mito/autophagy markers observed under acute FO conditions was normalized by 10 weeks, suggesting a cellular environment favoring mitochondrial biogenesis to accommodate the aerobic demands of the plantaris muscle. We also observed a significant increase in the expression of mitochondrial-, but not nuclear-, encoded oxidative phosphorylation (OXPHOS) proteins and peptides (i.e., humanin and MOTS-c) under chronic, but not acute, FO conditions. Taken together, the temporal response of markers related to the mitochondrial life cycle indicates a pattern of promoting biogenesis and mitochondrial protein expression to support the energy demands and/or enhanced neural recruitment of chronically overloaded skeletal muscle.

## Introduction

Skeletal muscle hypertrophy is a complex adaptive event in response to an increase in the mechanical load placed on this tissue. Hypertrophy generally manifests as an increase in contractile protein expression and content that can be quantified by examining myosin heavy-chain (MHC) protein isoforms (Tsika et al., 1987). Concomitant with increases in MHC proteins, hypertrophy also augments the myonuclear number (Allen et al., 1995; Roy et al., 1999), capillarity (McCall et al., 1996; Egginton et al., 1998), and whole-muscle and fiber cross-sectional areas, including fibers that are phenotypically slow, fast, and of mixed composition (Roy et al., 1997; Smith et al., 2002). In the rodent plantaris muscle, 3–10 weeks of chronic functional overload (FO) after surgical removal of hind limb synergists are associated with a shift in the MHC isoform composition, including a general increase in slower-contracting (types I and IIa) and a decrease in fast-contracting (type IIb) MHC isoforms (Tsika et al., 1987; Sugiura et al., 1993; Fauteck and Kandarian, 1995; Dunn and Michel, 1997; Roy et al., 1997; Bigard et al., 2001; Smith et al., 2002). Similar phenotypic shifts have been observed in human muscle following intense resistance training protocols, albeit to a lesser degree than detected with rodent paradigms of intermittent or chronic loading (Staron et al., 1990; Adams et al., 1993; Staron et al., 1994; McCall et al., 1996; Kadi and Thornell, 1999; Putman et al., 2004).

The fast-to-slow contractile shift occurring in hypertrophied skeletal muscle implies that the adapted muscle also increases the interfibrillar or subsarcolemmal mitochondrial volume, given that a greater mitochondrial density is typically associated with slow versus fast muscle phenotypes (Ingjer, 1979; Kirkwood et al., 1986; Philippi and Sillau, 1994; Takekura et al., 1994). However, early evidence from enzymatic or electron microscopy approaches does not support this paradigm, showing no change or a decrease in the mitochondrial volume in hypertrophic skeletal muscle (Lüthi et al., 1986; MacDougall et al., 1979; for review, see Mesquita et al., 2021). More recent work has revealed that strength/resistance training improves oxidative capacity in human muscle despite no changes in mitochondrial mass or expression of markers associated with mitochondrial biogenesis (Porter et al., 2015). Resistance training also appears to alter mitochondrial morphology: although strength-trained individuals have a similar mitochondrial volume density compared to untrained controls, they demonstrate an increased mitochondrial cristae density and surface-to-volume ratio despite a decrease in the overall mitochondrial size (Botella et al., 2023). Collectively, although the volume, or density, of skeletal muscle mitochondria does not appear to parallel the fast-to-slow MHC isoform shifts with hypertrophy, the changes in the quality of this organelle seem to reflect aerobic enhancements that coincide with these MHC phenotype changes (Nielsen et al., 2016; Parry et al., 2020; Memme et al., 2021) and are generally associated with a greater capacity for oxidative metabolism in more chronically active skeletal muscles.

To examine the mitochondrial responses to hypertrophic stimuli, we examined key signaling and regulatory markers of the mitochondrial life cycle within FO plantaris muscle, including markers associated with mitochondrial biogenesis, oxidative phosphorylation (OXPHOS), fission/fusion, and mito/autophagy (Memme et al., 2021). We examined these responses after 1 week of FO, i.e., before a marked change in muscle size and/or phenotypic shifts were expected, and also after 10 weeks of FO, when the hypertrophy and the fast-to-slow MHC adaptations had predominantly occurred (Tsika et al., 1987). We hypothesized that these mitochondrial biomarkers would indicate active biogenesis at 1 week, during the acute FO hypertrophy state, which would then subside after 10 weeks of FO. With continued chronic loading after 10 weeks of FO, we hypothesized that OXPHOS markers would increase compared to 1 week, reflecting the expected phenotypic shift to a more aerobic (e.g., type I MHC) profile. Finally, we expected a persistent upregulation of proteins associated with mito/autophagy during FO, reflecting the ongoing cellular maintenance of the adapting FO plantaris muscle.

## Methods

The experimental and animal care procedures described here were approved by the University of Puget Sound (Tacoma, WA) and followed the guidelines of the American Physiological Society. After a 48-h acclimatization period following arrival, adult female Sprague–Dawley rats (∼225 g) were assigned randomly to one of five groups: 0- (n = 5) and 10-week (n = 8) controls (CON) or experimental groups of either 3 days (n = 5), 7 days (n = 5), or 10 weeks (n = 9), following the surgical intervention to induce hypertrophy of the plantaris muscle by bilateral FO after the surgical removal of the gastrocnemius muscle (Baldwin et al., 1982). The soleus muscle was left intact but was not analyzed for the present study in as much as this muscle is nearly entirely slow (e.g., type I MHC); thus, any phenotypic or mitochondrial adaptations would likely be subtle and difficult to detect compared to the plantaris muscle, as observed earlier (Hyatt et al., 2019). The rats were euthanized with an overdose of sodium pentobarbital (100 mg/kg), and the plantaris was removed bilaterally, trimmed of excess fat and connective tissue, wet weighed, frozen in isopentane cooled by liquid nitrogen, and stored at −80°C until further analysis.

### Protein isolation and myosin heavy-chain isoform separation

Skeletal muscle portions near the mid-belly (∼50–80 mg) were homogenized using a motorized glass mortar and pestle in an 10-volume ice-cold buffer (pH 6.8) containing 10 mM Tris-HCl, 5 mM EDTA, 0.25 M sucrose, 100 mM KCl, 0.5% Triton-X, and a protease and phosphatase inhibitor cocktail (PPC1010; Sigma, St. Louis, MO) containing 4-(2-aminoethyl)benzenesulfonyl fluoride hydrochloride, aprotinin, bestatin hydrochloride, N-(trans-epoxysuccinyl)-L-leucine 4-guanidinobutylamide, leupeptin, pepstatin A, cantharidin (−)-p-bromolevamisole oxalate, and calyculin A. After homogenization, the samples were separated into two aliquots for MHC or total protein isolations. The MHC aliquot was centrifuged at 1,000 *g* for 10 min at 4°C, and the pellet was resuspended in an ice-cold wash buffer (10 mM Tris-HCl, 2 mM EDTA, 175 mM KCl, and 0.5% Triton-X at pH 6.8) and centrifuged again at 1,000 *g* for 5 min at 4°C. Finally, the pellet was washed in the ice-cold buffer (10 mM Tris-HCl and 150 mM KCl at pH 7.0), centrifuged at 1,000 *g* for 5 min at 4°C, and resuspended in 50 μL of the same buffer. The total protein aliquot was transferred to clean tubes immediately following homogenization and centrifuged at 12,000 *g* for 10 min at 4°C. The supernatant was transferred to clean tubes in 50-μL aliquots and frozen at −80°C for Western blot analyses. The MHC and total protein concentrations were determined using the Bio-Rad Protein Assay (Bio-Rad, Hercules, CA), according to the manufacturer’s instructions.

MHC isoforms were separated using standard methods outlined by Talmadge and Roy (1993). In brief, MHC isolates from each sample were boiled for 2 min at 100°C in a sample buffer, and 12.5 μg of each sample was loaded into wells of a 1.0-mm-thick 8% SDS-PAGE gel (using acrylamide:bis-acrylamide at a ratio of 29:1) that was then subjected to 65 V for 1 h, followed by 85 V for 22–24 h at 4°C. Following electrophoresis, the proteins were fixed in 12.5% trichloroacetic acid for 10 min and rinsed in ddH_2_O, and the gels were stained with quick Coomassie blue R-250 (Sigma) for 1 h and destained with ddH_2_O. Each gel was scanned, and the separated MHC bands were quantified using ImageJ (Rasband, 2024). Each MHC isoform was expressed as a percent of the total MHC protein detected for the sample.

### Western blot analysis

The following primary antibodies were used for Western blotting (source [e.g., company], catalog number, and the dilution used are included): total AMP-activated protein kinase α (AMPK; Cell Signaling, Inc., Danvers, MA; #2532; 1:2,000); phosphorylated AMPKα (Thr_172_) (pAMPK; Cell Signaling, Inc.; #2535; 1:1,000); nuclear respiratory factor 1 (NRF1; Abcam, Cambridge, MA; ab175932; 1:1,750); nuclear respiratory factor 2 (NRF2; Abcam; ab31163; 1:1,000); PPAR-gamma coactivator 1-alpha (PGC-1α; Abcam; ab54481; 1:1,000); phosphorylated PGC1⍺ (S_571_) (pPGC-1⍺; R&D Systems, Inc., Minneapolis, MN; AF6650; 1:1,000); sirtuin 1 (SIRT1; Abcam; ab110304; 1:1,000); mitochondrial transcription factor A (TFAM; Abcam; ab131607; 1:2,000); an OXPHOS antibody cocktail for the ATP synthase subunit of complex V (ATP5A), mitochondrially encoded cytochrome C oxidase I (MTCO1) of complex IV, core protein 2 of the ubiquinol–cytochrome C reductase (UQCRC2) in complex III, succinate dehydrogenase subunit (SDHB) in complex II, and NADH dehydrogenase subunit (NDUFB8) in complex I (Abcam; ab110413; 1:1,000); cytochrome C oxidase III (MTCO3; Proteintech, Rosemont, IL; 55082-1-AP; 1:1,000); cytochrome oxidase B (CYTB, Proteintech; 55090-1-AP; 1:1,000); NADH dehydrogenase, subunit 1 (ND1; Proteintech; 19703-1-AP; 1:1,000); mitochondrially encoded ATP synthase 6 (ATP6; Proteintech, 55313-1-AP; 1:1,000); mitochondrial ORF of the 12S rRNA type-C (MOTS-c; rabbit anti-rat custom; 1:3,000); humanin (HN; Sigma; #H2414; 1:1,200); mitofusion 2 (Mfn2; Abcam; ab124773; 1:1,000); mitochondrial fission protein 1 (Fis1; Abcam; ab96764; 1:1,000); dynamin-related protein 1 (Drp1; Abcam; ab56788; 1:2,500); mitochondrial fission factor (MFF; Cell Signaling Inc.; 84580S; 1:1,000); PTEN-induced kinase 1 (PINK1; Abcam; ab23707; 1:750); parkin (Abcam; ab77924; 1:1,000); total TANK-binding kinase 1 (TBK1; Cell Signaling Inc.; #3504; 1:1,000); phosphorylated TBK1 (Ser_172_) (pTBK1; Cell Signaling Inc.; #5483; 1:1,000); ubiquitin-binding protein p62 (p62; Abcam; ab56416; 1:1,500); and microtubule-associated proteins 1A/1B light-chain 3B (LC3-I and II; Abcam; ab128025; 1:2,000).

Loading of total protein for immunoblotting ranged between 10 and 50 μg, depending on the target (Hyatt et al., 2023). The total protein was separated using either 10, 12, or 15% acrylamide Tris-glycine gels. Samples were mixed in 2× sample buffer (0.2% SDS, 20% glycerol, 25% 4× buffer, 5% β-mercaptoethanol, and 0.025% bromophenol blue) and heated to either 42.5°C (if probing for MTCO1) or 100°C for 3 min and electrophoresed at 80 V for 20 min and then 140 V for 60–70 min. The proteins were wet-transferred to PVDF membranes for 100 min at 50 V for any target <20 kDa or for 3 h at 60 V. The membranes were then placed in a solution of Ponceau S (Sigma) to verify that the transfer was uniform and artifact-free, partially destained in ddH2O, and digitally scanned for sample loading control used in subsequent analyses and quantification. The membranes were then placed in 5% nonfat dry milk (NFM) dissolved in Tris-buffered saline with 0.05% Tween-20 (T-TBS) for a minimum of 0.5 h. The membranes were incubated in a primary antibody diluted in NFM for 1 h at room temperature (RT) or overnight at 4°C. Following serial washes, the membranes were incubated in an NFM-diluted species-appropriate IgG-HRP-conjugated secondary antibody (Jackson ImmunoResearch Labs, Inc.; 1:1,000–10,000) for 1 h at RT (Hyatt et al., 2023). Finally, the membranes were developed using ECL detection. Densitometry and quantification were performed using ImageJ software (Rasband, 2024).

### Immunohistochemistry

Immunohistochemistry was performed to qualitatively confirm phenotypic shifts in FO muscles and test the hypothesis that mitochondrial ORF of the 12S rRNA type-C (MOTS-c) is concentrated in predominantly slow fibers because of the inherent greater mitochondrial density in slow versus fast muscles (Hyatt, 2022; Kumagai et al., 2022; D’Souza et al., 2020). Serial cross-sections (10-μm thick) of plantaris muscles were air-dried and fixed for 15 min in a fresh 4% formaldehyde solution and then washed in three successive rinses of 1× phosphate-buffered saline (PBS). To quench endogenous peroxidases, samples were incubated for 30 min in a 0.3% H_2_O_2_ solution. Following several rinses in PBS, the samples were blocked in 5% normal goat serum (NGS) for 15 min and then incubated for 1 h at RT in either a mouse anti-slow MHC (Sigma; M8421; 1:50) or a custom rabbit anti-MOTS-c (1:25 dilution) primary antibody diluted in 1× PBS. After three successive rinses in PBS, the samples were blocked in NGS for 5 min, followed by incubation in either an anti-mouse or -rabbit-HRP-conjugated secondary antibody for 1 h at RT (1:75 dilution). For staining controls, the samples were incubated in PBS instead of the primary antibody, followed by incubation in either secondary antibody. Following three successive rinses in PBS, the samples were developed for 2–5 min in a 3,3′-diaminobenzidine solution containing 0.1% v/v H_2_O_2_. Samples were serially dehydrated, cleared, mounted in Permount medium, and qualitatively assessed.

### Statistics

The values are presented as the mean ± SE. One-way analysis of variance (ANOVA) was performed to compare the means for each variable for the CON and acute FO groups (CON and FO 3 days or FO 7 days). For *post hoc* comparisons between the means, Student’s one-tailed *t*-test using a Bonferroni correction was performed for changes in muscle mass, and a two-tailed *t*-test was used for biochemical markers. For chronic FO (CON and FO 10 weeks), one- and two-tailed Student’s *t*-tests were used for muscle mass and biochemical markers, respectively. SPSS (v24) was used for statistical analysis, with significance set at *p* < 0.05; however, differences of *p* < 0.01 and trends (*p* = 0.06–0.1) were noted throughout.

## Results

The absolute mass of the plantaris did not increase until 7 days after FO surgery (Table 1). The FO 7-day plantaris mass was 11.8% and 10.9% greater (*p* < 0.05) than that of CON or FO 3-day muscles, respectively, whereas the muscle mass relative to body weight trended greater at 7 days than that of CON (*p* = 0.06) and FO 3-day (*p* = 0.07) muscles. By 10 weeks, the absolute and relative plantaris muscle masses were 26.3% and 25.8% greater, respectively, than those of the aged-matched controls (*p* < 0.05).

**TABLE 1:** Body and plantaris muscle masses following acute and chronic functional overload.

		Plantaris Mass		
		Body mass (g)	Absolute (mg)	Relative (mg/g)
**Acute**	**CON**	224.4 ± 2.2	263.0 ± 8.5	1.17 ± 0.03
	**FO 3d**	226.4 ± 3.1	265.3 ± 7.3	1.17 ± 0.03
		*(0.9)*	*(0.9)*	*(0.003)*
	**FO 7d**	233.4 ± 3.9	300.76.4*+	1.22 ± 0.03
		*(3.0)*	*(11.8)*	*(4.22)*
**Chronic**	**CON**	261 ± 2.8	295.6 ± 5.5	1.13 ± 0.02
	**FO 10 wk**	262.3 ± 6.2	401.0 15.9 *	1.53 ± 0.05*
		*(0.5)*	*(26.3)*	*(25.8)*

*: different from within group CON (*p* <0.05); † : different from FO 3d: different from CON and FO 3d (*p* <0.1). *italicized values* = % change from acute or chronic CON group, respectively.

Note: “CON” in acute and chronic conditions are distinct, age-matched cohorts.

**FIGURE 1:**
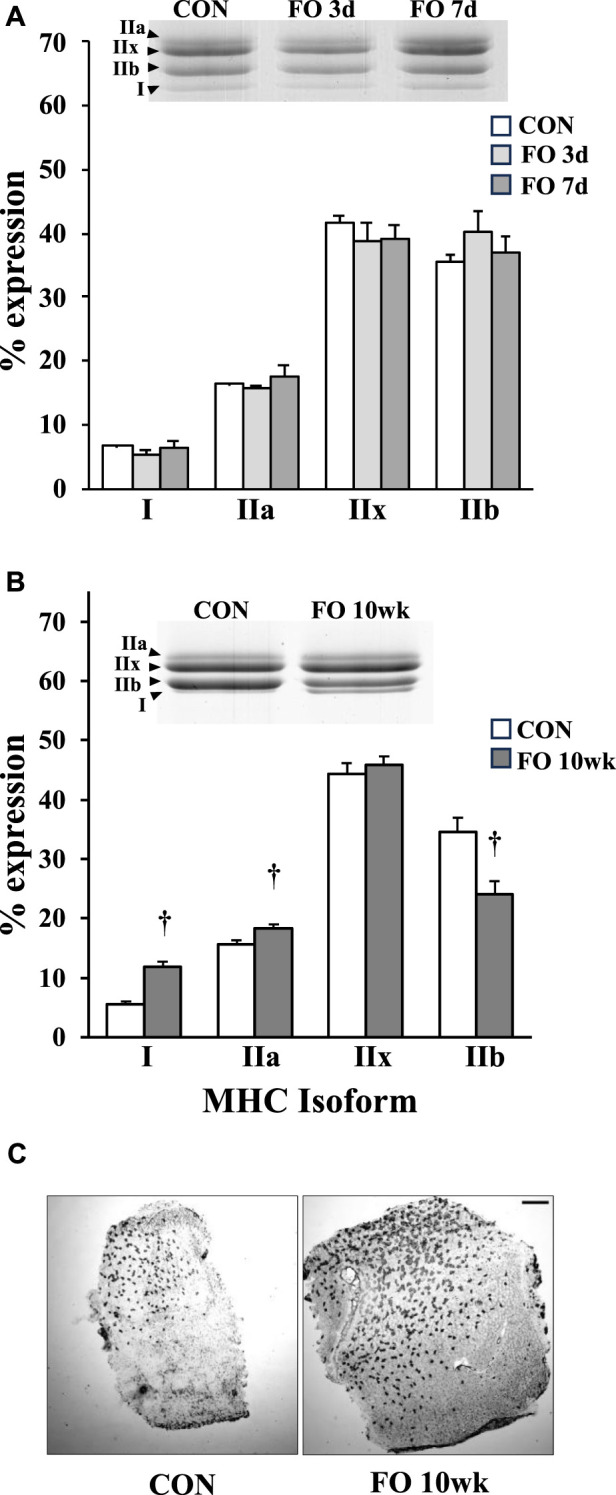
Percent myosin heavy-chain isoform expression in acute **(A)** and chronic **(B)** FO plantaris muscles of adult female rats. FO was accomplished by the synergistic ablation of the gastrocnemius muscles, followed by a recovery period of 3 days (3 d), 7 days (7 d), or 10 weeks (10 wk). Control (CON) rats for acute (≤1 week) and chronic (10 weeks) time points were assigned to distinct, aged-matched groups to account for any age-related MHC shifts. For each sample, the percentage of each isoform relative to the total MHC content was determined. The increase in type I MHC phenotype at 10 weeks was confirmed qualitatively using immunohistochemistry **(C)**. Scale bar = 0.5 mm. Values show the mean ± SE. †: significantly different from age-matched within-group CON; *p* < 0.01.

There were no significant phenotypic shifts in MHC protein content in acute FO (3 days or 7 days) muscles compared to age-matched controls (Figure 1; *p* > 0.05). By 10 weeks, type I MHC content in FO plantaris muscles increased by 2.1-fold compared to age-matched controls, whereas type IIa exhibited a 17% increase from controls (*p* < 0.01). In contrast, the content of type IIb MHC decreased by ∼30% in FO 10-week plantaris muscles relative to CON (*p* < 0.01). This fast-to-slow phenotypic shift was verified histochemically in type I MHC-stained FO 10-week muscles (Figure 1).

**FIGURE 2:**
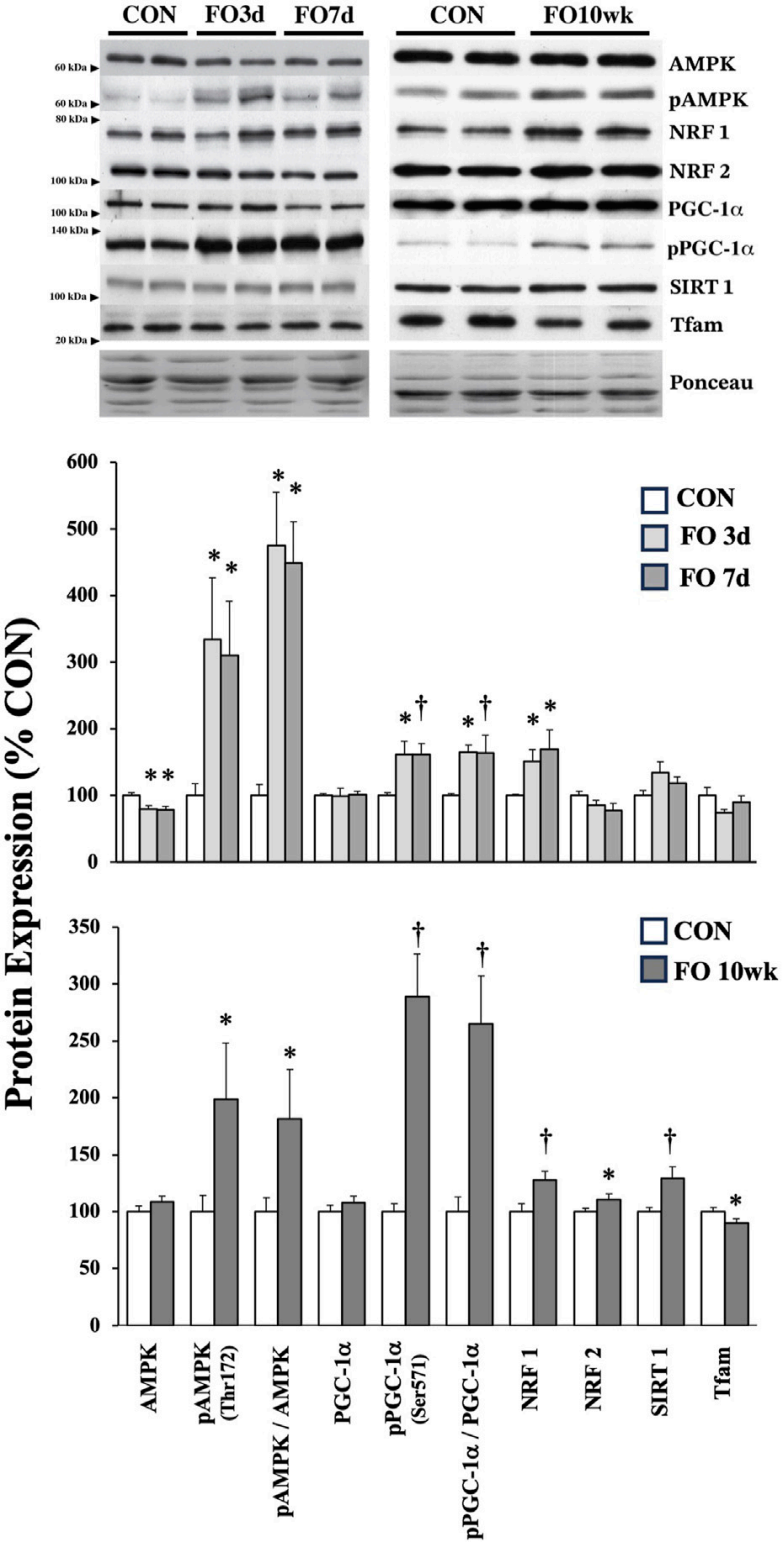
Expression of protein biomarkers associated with mitochondrial biogenesis in acute (*top*) and chronic (*bottom*) FO plantaris muscles of adult female rats. FO was accomplished by the synergistic ablation of the gastrocnemius muscles, followed by a recovery period of 3 days (3 d), 7 days (7 d), or 10 weeks (10 wk). CON rats for acute (≤1 week) and chronic (10 weeks) time points were assigned to distinct, aged-matched groups. A representative blot for each target is shown. Values show the mean ± SE. *: significantly different from within-group CON, *p* < 0.05; †: significantly different from within-group CON, *p* < 0.01.

Protein markers of mitochondrial biogenesis were assessed in acute and chronic FO muscles (Figure 2). Despite a decrease in the total AMPK expression by ∼20% in FO 3-day and 7-day plantaris muscles (*p* < 0.05), the pAMPK level was elevated 3.3- and 3.1-fold, respectively, relative to CON (*p* < 0.05). The pAMPK level remained elevated in FO plantaris muscles ∼2-fold after 10 weeks (*p* < 0.05). NRF1 expression significantly increased by ∼50 (*p* < 0.05), 70 (*p* < 0.05), and 27% (*p* < 0.01) in FO 3-day, 7-day, and 10-week plantaris muscles compared to aged-matched controls. The NRF2 level, however, was modestly elevated (∼11%; *p* < 0.05) in FO 10-week muscles only. The pPGC-1α level was elevated by ∼60% in FO 3-day and 7-day (*p* < 0.05) and 2.9-fold in FO 10-week (*p* < 0.01) plantaris muscles. At 10 weeks, FO plantaris muscles exhibited a 28% increase in SIRT1 (*p* < 0.01) and a small, but significant, decrease (10%; *p* < 0.05) in TFAM expression.

**FIGURE 3:**
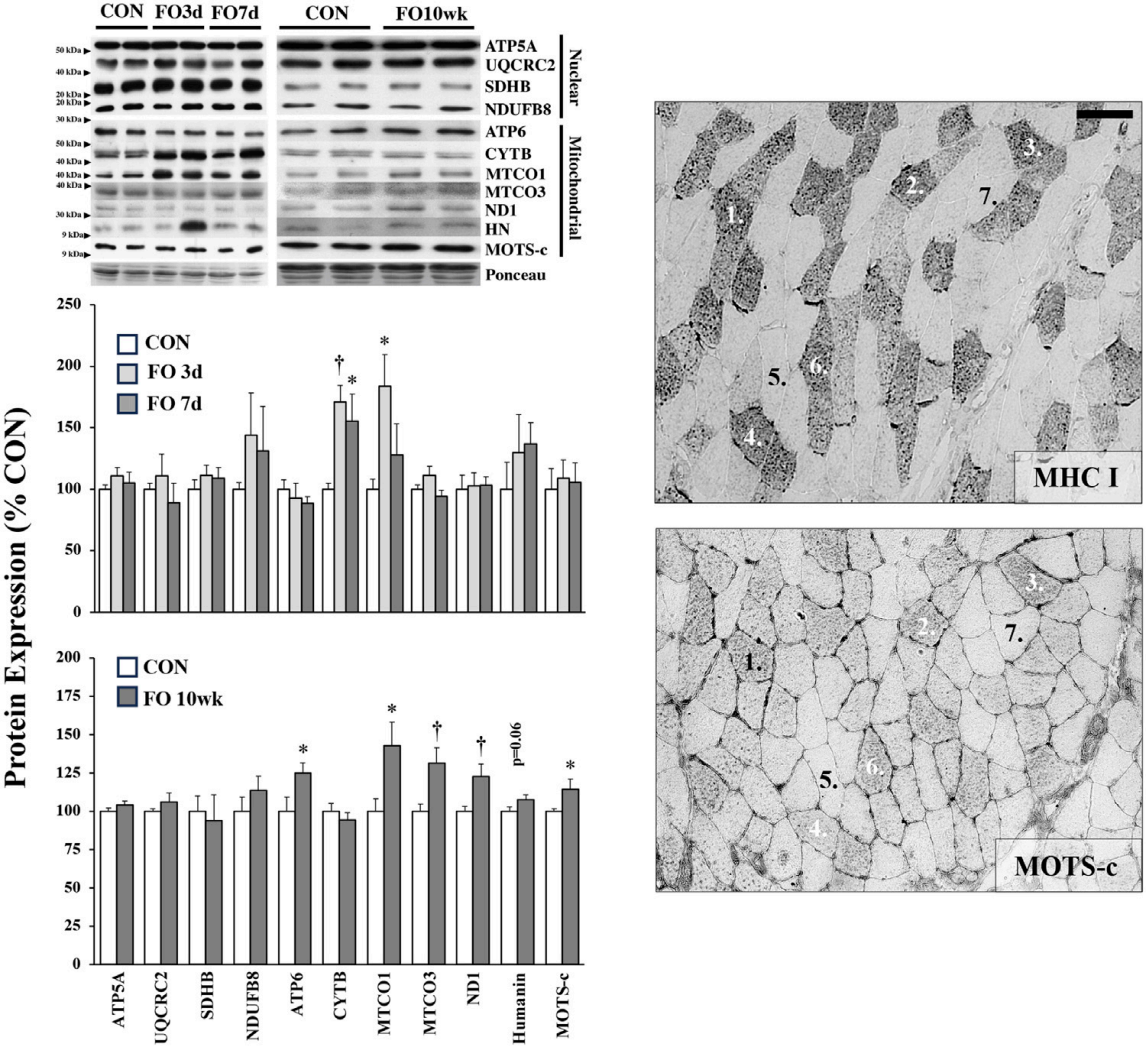
Expression of nuclear- and mitochondrial-encoded proteins in acute (*left, top*) and chronic (*left, bottom*) FO plantaris muscles of adult female rats. FO was accomplished by the synergistic ablation of the gastrocnemius muscles, followed by a recovery period of 3 days (3 d), 7 days (7 d), or 10 weeks (10 wk). CON rats for acute (≤1 week) and chronic (10 weeks) time points were assigned to distinct, aged-matched groups. All targets are subunits associated with various complexes of the electron transport chain/oxidative phosphorylation except for the peptides humanin (HN) and mitochondrial ORF of the 12S rRNA type-c (MOTS-c). A representative blot for each target is shown. MOTS-c colocalized with type I MHC fibers in serial muscle cross-sections (*right*). Scale bar = 20 μm. Values show the mean ± SE. *: significantly different from within-group CON, *p* < 0.05; †: significantly different from within-group CON, *p* < 0.01.

The expression of mitochondrial-specific proteins in acute and chronic FO muscle is shown in Figure 3. No changes were observed in the expression of nuclear-encoded mitochondrial proteins associated with oxidative phosphorylation complexes, including ATP5A, UQCRC2, SDHB, and NDUFB8. In contrast, mitochondrial DNA (mtDNA)-encoded proteins generally increased in response to FO, particularly at the 10-week time point. Compared to CON, the expression of CYTB was elevated 71% and 55% in FO 3-day (*p* < 0.01) and 7-day (*p* < 0.05) muscles, respectively. MTCO1 expression increased by ∼11% in FO 3-day (*p* < 0.05) but was unchanged in FO 7-day plantaris muscles. Compared to CON, an increase in the expression of ATP6 (24%), MTCO1 (42%), MTCO3 (31%), ND1 (22%), and MOTS-c (14%) was observed in FO 10-week muscles. A trend in elevated HN expression was also detected in FO 10-week plantaris muscles (*p* = 0.06). Finally, MOTS-c protein expression localization coincided with fibers that were also identified as positive slow (type I) MHC (Figure 3).

**FIGURE 4:**
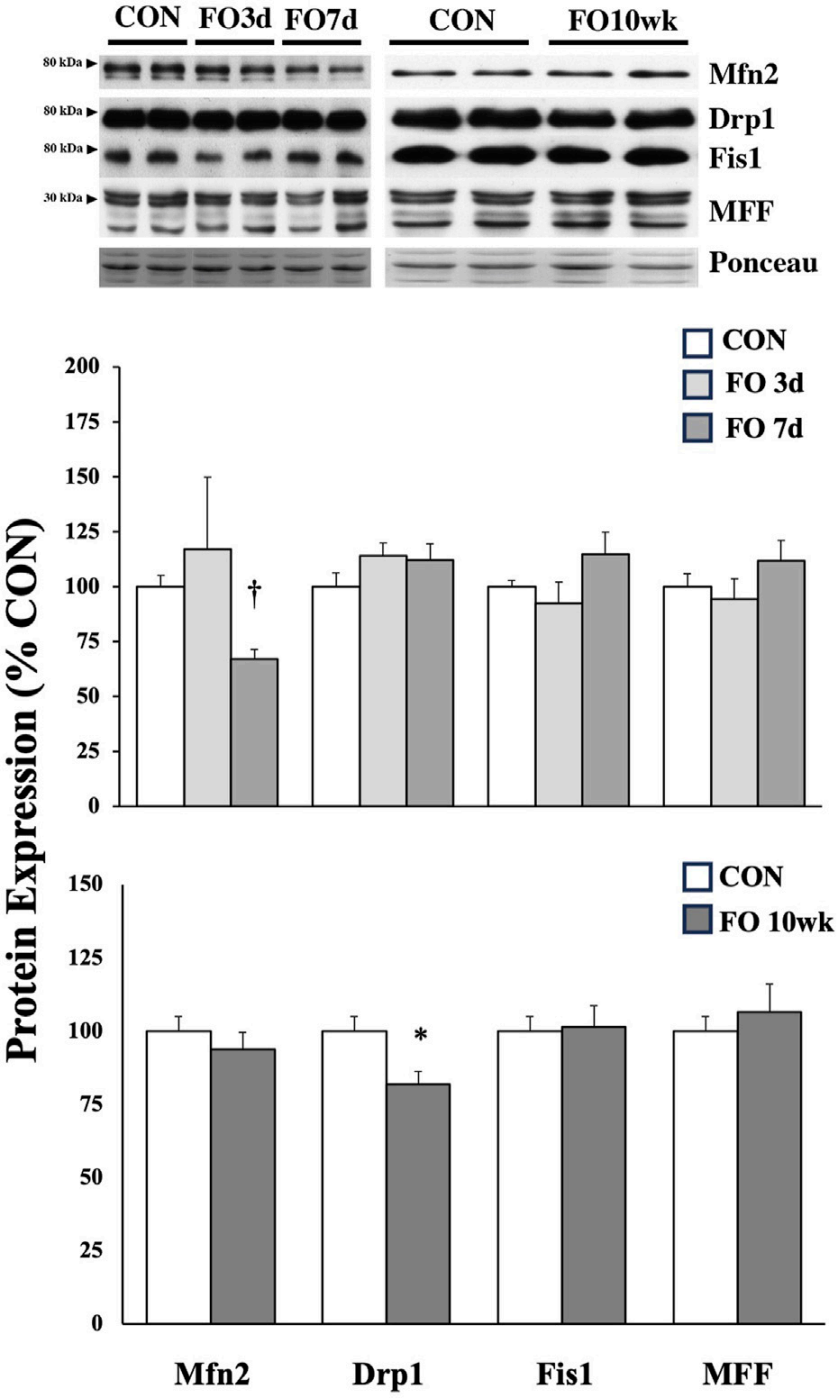
Expression of protein biomarkers associated with mitochondrial fusion (Mfn2) or fission in acute (*top*) and chronic (*bottom*) FO plantaris muscles of adult female rats. FO was accomplished by the synergistic ablation of the gastrocnemius muscles, followed by a recovery period of 3 days (3d), 7 days (7 d), or 10 weeks (10 wk). CON rats for acute (≤1 week) and chronic (10 weeks) time points were assigned to distinct, aged-matched groups. A representative blot for each target is shown. It should be noted that the antibody for the mitochondrial fission factor (MFF) protein tags four isoforms, which migrate similarly through the gel (25, 27, 30, and 35 kDa); for analysis, MFF isoforms were collectively scanned, quantified, and compared between groups. Values show the mean ± SE. *: significantly different from within-group CON, *p* < 0.05; †: significantly different from within-group CON, *p* < 0.01.

Proteins associated with fission and fusion of mitochondria were mostly unchanged under acute or chronic FO conditions (Figure 4). Compared to aged-matched controls, Mfn2 expression was ∼33% lower in FO 7-day muscles (*p* < 0.01), whereas the Drp1 expression decreased by ∼18% in the FO 10-week plantaris (*p* < 0.05).

**FIGURE 5:**
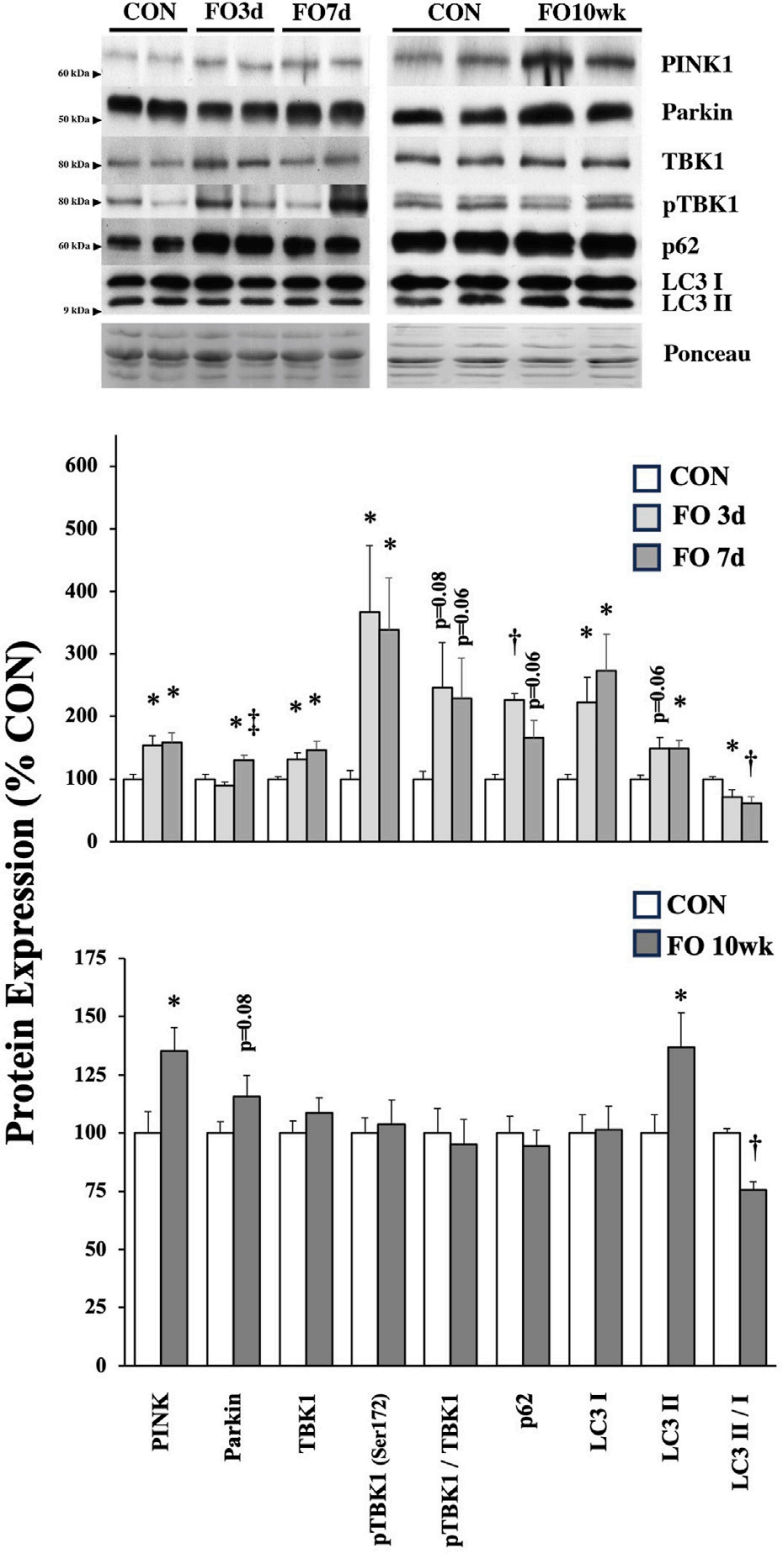
Expression of protein biomarkers associated with mitophagy/autophagy in acute (*top*) and chronic (*bottom*) FO plantaris muscles of adult female rats. FO was accomplished by the synergistic ablation of the gastrocnemius muscles, followed by a recovery period of 3 days (3 d), 7 days (7 d), or 10 weeks (10 wk). CON rats for acute (≤1 week) and chronic (10 weeks) time points were assigned to distinct, aged-matched groups. A representative blot for each target is shown. Values show the mean ± SE. *: significantly different from within-group CON, *p* < 0.05; †: significantly different from within-group CON, *p* < 0.01. ‡: significantly different from FO 3-day muscle.

The expression of proteins associated with cellular degradation and recycling (e.g., mito/autophagy) was assessed in FO muscles (Figure 5). PINK1 expression increased 53, 58, and 35% in 3-day, 7-day, and 10-week FO muscles, respectively (*p* < 0.05). Parkin protein expression in FO 7-day muscle increased significantly from CON (*p* < 0.05) and FO 3-day (*p* < 0.01) levels, whereas parkin expression trended higher (*p* = 0.08) in FO 10-week muscles than that in aged-matched controls. Total TBK1 and pTBK1 levels were elevated 31% and 3.6-fold in FO 3-day and 46% and 3.4-fold in FO 7-day muscles (*p* < 0.05), respectively, but unchanged at 10 weeks. Compared to CON, p62 expression increased 2.2-fold in FO 3-day plantaris muscles (*p* < 0.01), trended (*p* = 0.06) higher at 7 days, and was unchanged at 10 weeks. LC3-I expression was elevated 2.3- and 2.7-fold at 3 and 7 days, respectively (*p* < 0.05). LC3-II expression was significantly greater in FO 7-day and 10-week plantaris muscles (*p* < 0.05) and trended (*p* = 0.06) higher at 3 days. The LC3-I/-II ratio was significantly lower in plantaris muscles in all FO groups (*p* < 0.05).

## Discussion

The goal of this study was to capture, in part, the temporal expression patterns for markers of the phases of the mitochondrial life cycle during early and late time points of skeletal muscle hypertrophy (Tsika et al., 1987). Unexpectedly, we found that the expression of proteins associated with mitochondrial biogenesis was not only elevated during periods of acute overload (≤1 week) but also sustained after 10 weeks. Interestingly, the fast-to-slow phenotypic shift detected in 10-week FO muscles was paralleled by an increase in mitochondrial-, but not in nuclear-, encoded OXPHOS proteins. Furthermore, contrary to our hypothesis, we observed a general increase in markers attributed to mito/autophagy in 3- and 7-day FO muscles (e.g., FO 1 week), which mostly normalized after 10 weeks. Taken together, the skeletal muscle activates mechanisms to promote and maintain mitochondrial biogenesis during hypertrophy, presumably to match the shift to a more physiological aerobic capacity/phenotype.

The rat plantaris is a mixed muscle comprising ∼70–80% of fast (types IIx and IIb) MHC isoforms. In agreement with earlier work, we show that the long-term overload of the plantaris muscle increases in slow (types I and IIa) MHC content (Figure 1), although the magnitude of this fast-to-slow shift, as well as the overall change in plantaris mass, appears dependent on the duration following FO surgery and whether the soleus is removed along with the gastrocnemius muscles during the FO procedure (Swoap et al., 1994; Fauteck and Kandarian, 1995; Dunn and Michel, 1997; Roy et al., 1997; Bigard et al., 2001). For example, because we left the soleus muscle intact in our model, we detected only a ∼25% increase in muscle mass after 10 weeks (Table 1); after a similar time period, plantaris muscle hypertrophy may exceed 55% from control levels with the removal of the soleus (Tsika et al., 1987; Swoap et al., 1994; Roy et al., 1997). We also found that MHC types I and IIa increased by 116% and 17%, respectively, in FO 10-week plantaris muscles after the removal of the gastrocnemius only (Figure 1); however, others have shown that these isoforms may increase ∼300% from control levels when all hind limb synergists are ablated (Tsika et al., 1987; Swoap et al., 1994; Dunn and Michel, 1997; Roy et al., 1997; Bigard et al., 2001). Consistent with other reports, we detected no changes in MHC isoforms at early (≤1 week) FO time points; phenotypic transition may take ∼9 weeks to stabilize (Tsika et al., 1987).

Overload of the plantaris muscle increases neural recruitment by ∼60–70% from control values within the first 2 weeks (Roy et al., 2011) and ∼200% from the baseline level after 2–4 weeks following FO surgery (Gardiner et al., 1986). This change in neuromuscular activity would disrupt intracellular homeostasis and activate signaling pathways, resulting in mitochondrial biogenesis (Wilkinson et al., 2008; Memme et al., 2021). This augmented activation in the FO 1-week plantaris muscle is reflected by the elevated activated/phosphorylated AMPK (pAMPK_Thr172_), likely resulting from an increase in the AMP:ATP ratio from elevated ATP hydrolysis or an increase in cytosolic calcium of recruited fibers (Kjøbsted et al., 2018). However, the pAMPK level remained elevated after hypertrophy and is known to slow down considerably in the FO 10-week plantaris muscles (Tsika et al., 1987), suggesting that disturbances to homeostasis and activation of AMPK are not exclusive to acute stimuli (Dreyer et al., 2006; Hamilton et al., 2014; Riedl et al., 2016; Shirai et al., 2020; Gombos et al., 2021). Here, we cannot specify whether activated AMPK is only directed toward maintaining the mitochondrial pool by activating mitochondrial biogenesis or for another divergent pathway such as mito/autophagy (Thomson, 2018; Seabright and Lai, 2020); at a minimum, these findings suggest that the activation of AMPK participates in maintaining the mitochondrial volume in chronic hypertrophy scenarios, possibly to match recruitment demands and shifts in the MHC phenotype.

The transcriptional co-activator, PGC-1α, is a well-known regulator of the skeletal muscle phenotype and mitochondrial biogenesis (Liang and Ward, 2006), as demonstrated in seminal work by Lin et al. (2002) and others (Leone et al., 2005; Mortensen et al., 2006; Rowe et al., 2013; Ballmann et al., 2016). Although total PGC-1α levels were unchanged in FO 1-week and 10-week muscles (Figure 2), we detected a ∼2–3-fold increase in phosphorylated (Ser_571_)/inactivated PGC-1α levels at each time point. The inactivation of PGC-1α at this site has been directly attributed to the control of gluconeogenesis and/or fatty acid oxidation via Akt (Li et al., 2007). Alternatively, because phosphorylation at this location (Ser_571_) lies in the serine/arginine-rich region of the C-terminus, an area of PGC-1α that directly interacts with transcription factors such as NRF1 (Villena, 2015), its phosphorylation could reflect a coordinated control of PGC-1α activity within the nucleus (Luo et al., 2019). Given that FO disrupts a multitude of homeostatic conditions that would, collectively, result in activating PGC-1α on other phosphorylation sites, there may be a need to instigate countermeasures modulating this hyperactivation to regulate the magnitude of PGC-1α-mediated transcription. Here, increases in NRF1/2 and/or SIRT1 protein expression may better signify ongoing mitochondrial biogenesis in FO 1-week and 10-week plantaris muscles, given that these proteins collectively influence the transcription of genes necessary for oxidative phosphorylation and mitochondrial membrane transport (Gurd, 2011; Tang, 2016; Gureev et al., 2019).

NRF1 is known to regulate the expression of nuclear-encoded (nDNA) OXPHOS genes (Evans and Scarpulla, 1990). Despite elevated NRF1 levels in FO 1-week and 10-week muscles, there was no change in nDNA OXPHOS proteins (Figure 3), but we observed an increase in mtDNA-encoded proteins, particularly at the 10-week time point. The observed increase in mtDNA-encoded proteins also occurred despite a small but significant decrease in TFAM protein expression in FO 10-week muscles, which is a known transcription factor for inducing mtDNA expression (Gordon et al., 2001). These observations may simply be because there is more mtDNA, or a scarcity of nDNA (Hansson et al., 2020), at 10 weeks than at 1 week, which parallels the observed phenotypic shifts (Figure 1). Indeed, type I fibers contain a greater mtDNA copy number per fiber area/volume than type IIa and IIb fibers (Durham et al., 2005; Herbst et al., 2017). However, given that long-term FO also increases the myonuclear number by ∼2- and 2.8-fold in slow and fast MHC fibers (Allen et al., 1995), respectively, there also appears to be an enhanced potential to express nDNA OXPHOS genes. The discrepancy between mtDNA and nDNA-encoded expression may be due to a blunted rate of nDNA transcription resulting from the inactivation of PGC-1α or limitations of nDNA-encoded protein transport into the mitochondria by translocase of the outer mitochondrial membrane (TOM) complex (Priesnitz and Becker, 2018; Gureev et al., 2019) and warrants further investigation. Humanin and MOTS-c, two mtDNA-encoded non-OXPHOS peptides, also exhibited small but significant increases in FO 10-week muscles. These mitokines have been shown to have both cellular and systemic impacts, particularly related to glucose–insulin dynamics, supporting the notion that skeletal muscle hypertrophy can, in part, assist with glycemic control (Lee et al., 2015; Gidlund et al., 2016; Kim et al., 2018; Reynolds et al., 2021; Sammut et al., 2024). At the whole-muscle level, higher MOTS-c protein expression is correlated with slow phenotypes (D’Souza et al., 2020; Hyatt, 2022), which is also manifested at the fiber-specific level (Figure 3). Recent work has even reported that MOTS-c may also influence the MHC phenotype (Kumagai et al., 2022), but this observation requires additional investigation.

Interestingly, we detected a greater expression of mito/autophagy-related proteins in FO 1-week than in 10-week muscles (Figure 5). Scervino et al. (2023) recently showed a general increase in autophagy-related proteins in the 7-day overloaded soleus and extensor digitorum longus (EDL), which is consistent with the notion that autophagy is required for early adaptive metabolic/oxidative events in response to exercise and/or augmented recruitment (Lira et al., 2013). It has been established that acute, or unaccustomed, exercise elevates the presence of reactive oxygen species (Leeuwenburgh et al., 1999; Sakai et al., 1999) and leads to mitochondrial dysfunction from altered calcium homeostasis (Hody et al., 2019), which would likely augment the mito/autophagy machinery necessary for cellular maintenance in FO 1-week muscles (Chatzinikita et al., 2023) but not at 10 weeks after homeostasis had been reestablished (Figure 5). With mito/autophagy normalized at 10 weeks, the conditions would favor mitochondrial biogenesis and, consequently, aerobic metabolism associated with a more oxidative muscle phenotype (Figures 1–3). Parkin overexpression has also been shown to increase mitochondrial content in 7-month-old mice, suggesting that elevated parkin expression in FO 7-day and 10-week plantaris muscles trends toward augmenting, rather than eliminating, the mitochondrial volume during hypertrophy (Leduc-Gaudet et al., 2019).

In summary, the expression of markers associated with the mitochondrial life cycle was not exclusive to acute periods during which the FO muscle experiences rapid adaptation and growth. Rather, adaptation associated with the mitochondria continued in overloaded plantaris muscle up to 10 weeks following synergistic ablation of hind limb muscles, particularly balanced toward mitochondrial biogenesis. Although our findings indicate a shift toward a more aerobic phenotype favoring mitochondrial expansion, further work is required to determine whether mitochondrial density/volume actually increases in hypertrophied muscle fibers. The notion of mitochondrial dilution, where the mitochondrial density/volume decreases relative to the quantity of myofibrillar proteins produced during hypertrophy, is a possible consequence of overall fiber enlargement (Groennebaek and Vissing, 2017; Parry et al., 2020).

## Data Availability

The raw data supporting the conclusion of this article will be made available by the authors, without undue reservation.
