# Supplementary figures and images for: Temporal expression of mitochondrial life cycle markers during acute and chronic overload of rat plantaris muscles
Source: Front Physiol. 2024 Aug 30;15:1420276. doi: 10.3389/fphys.2024.1420276 (PMC11392739; doi:10.3389/fphys.2024.1420276)

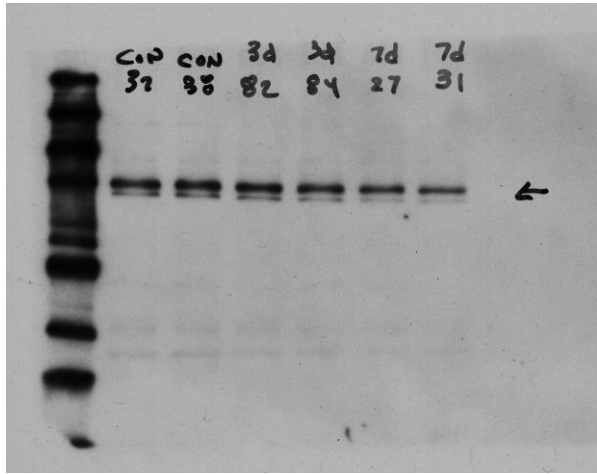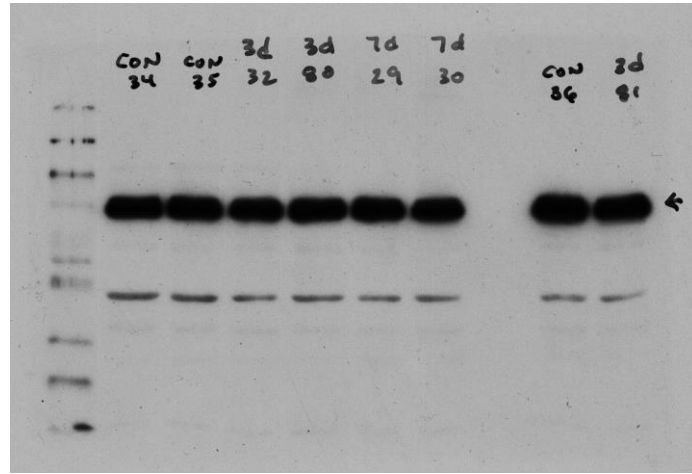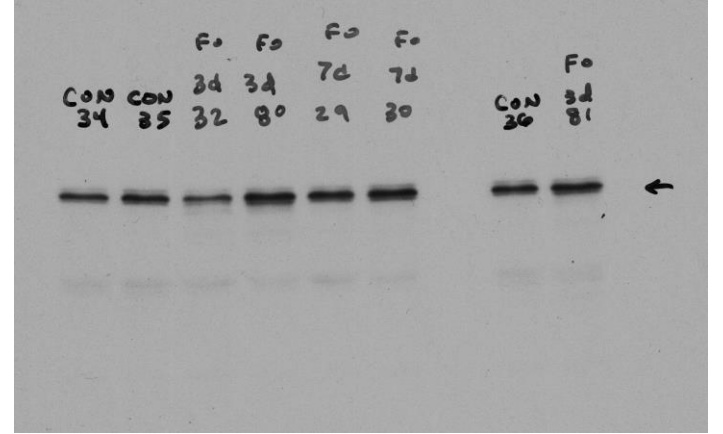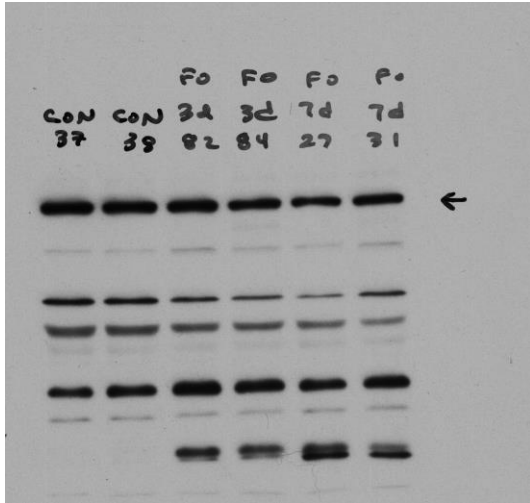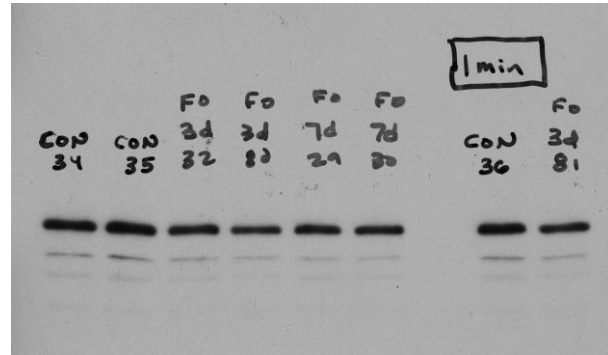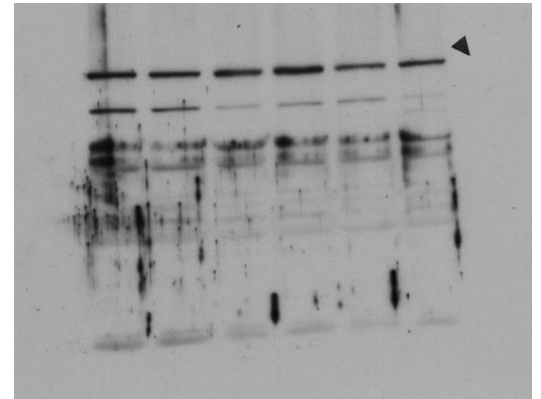

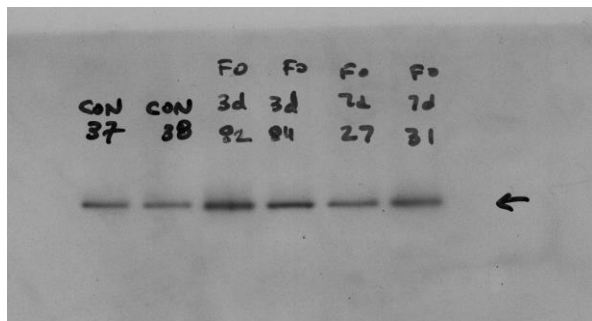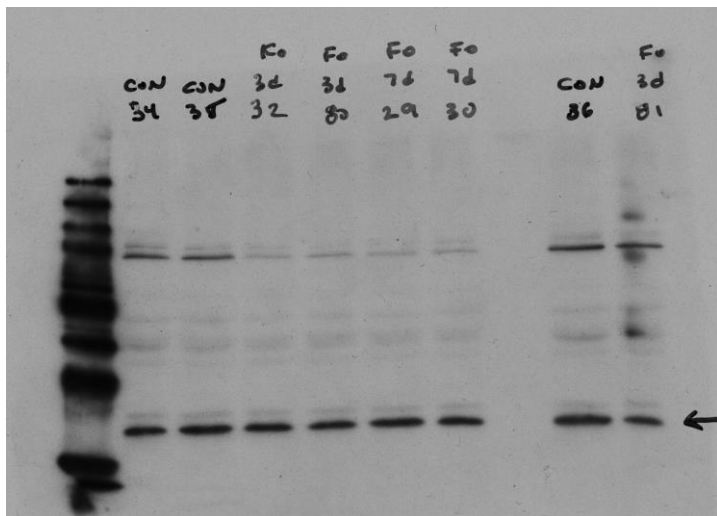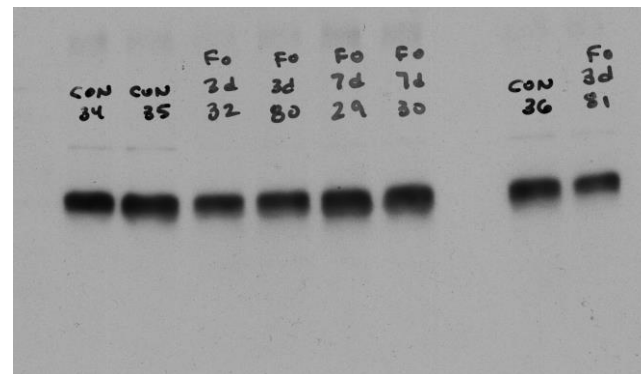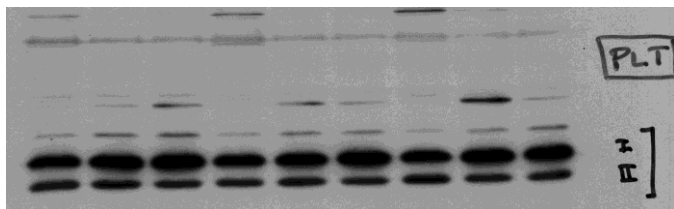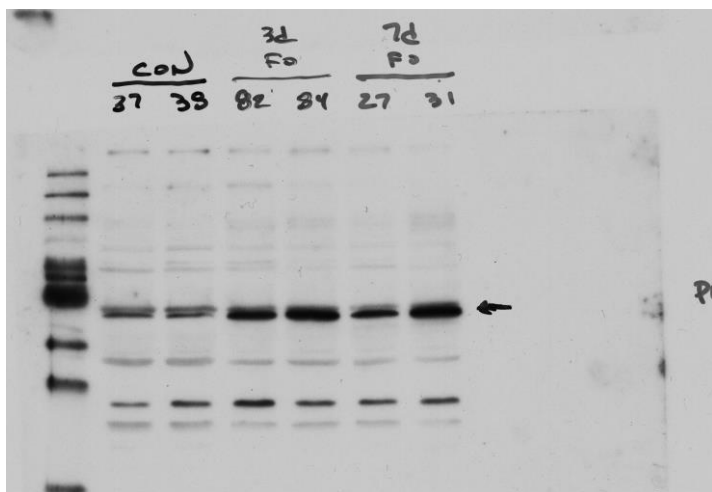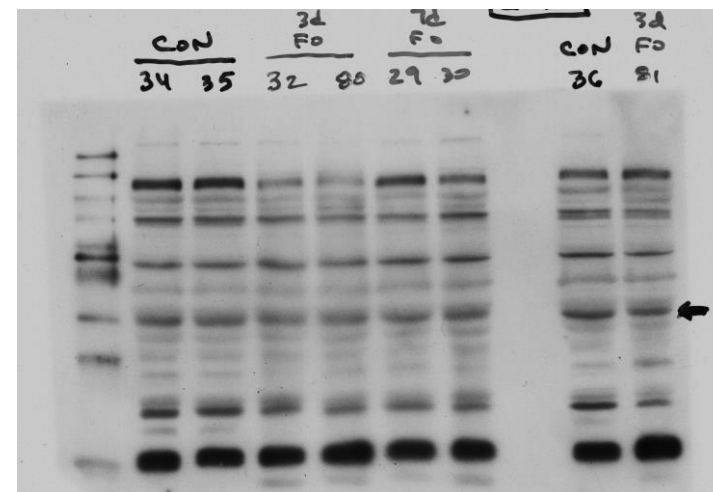

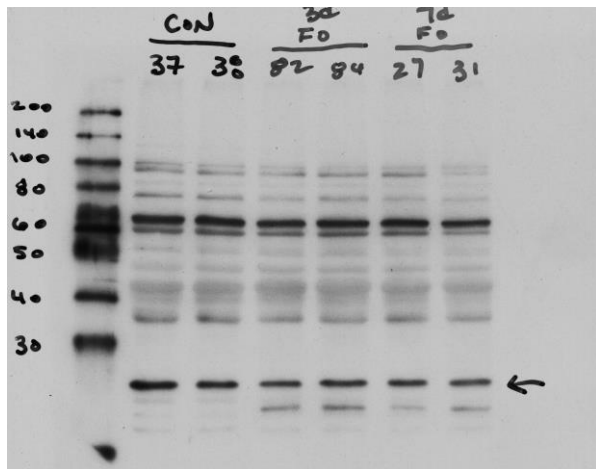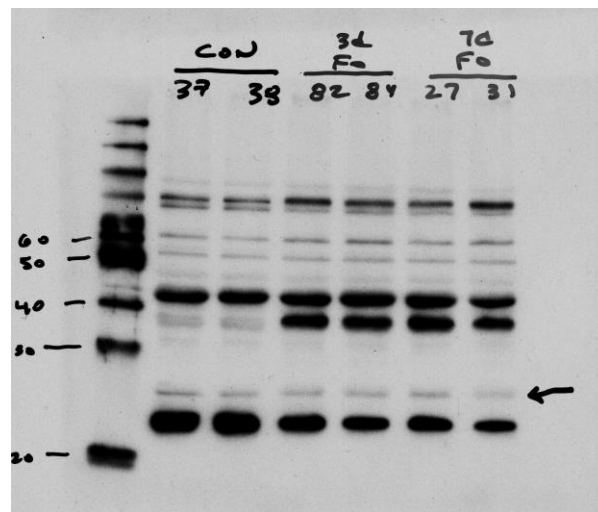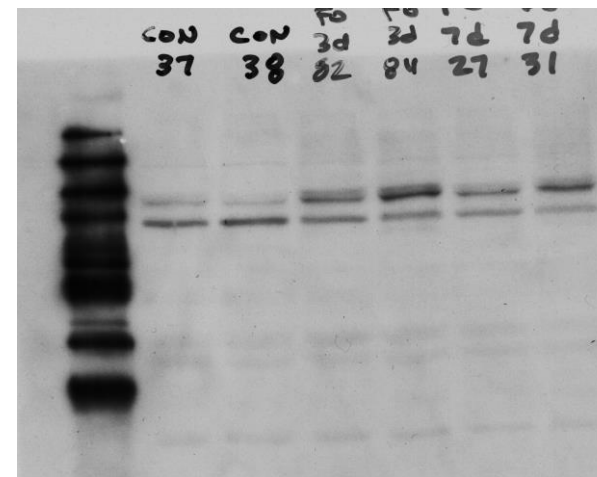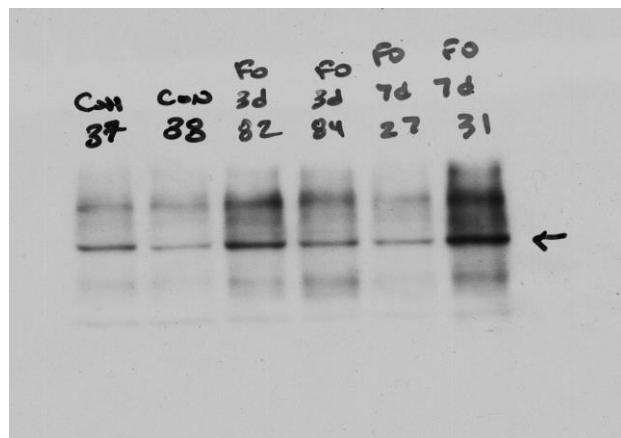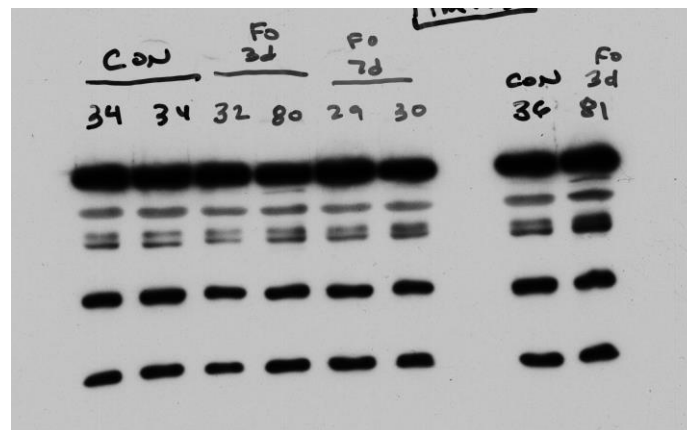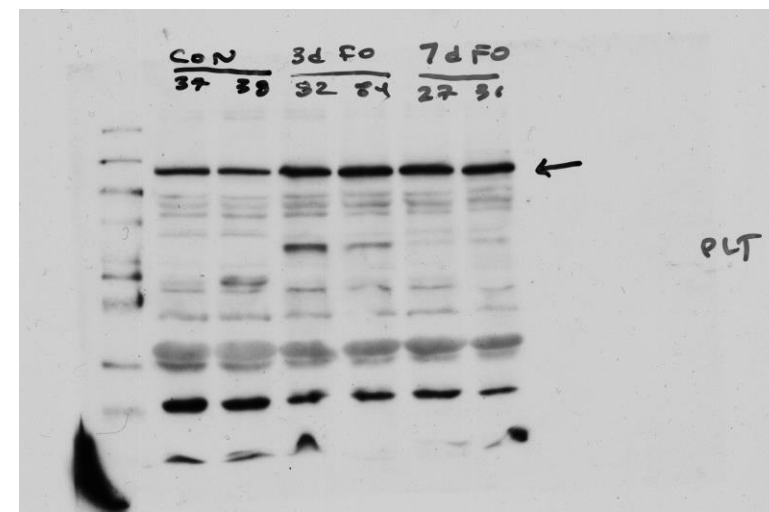

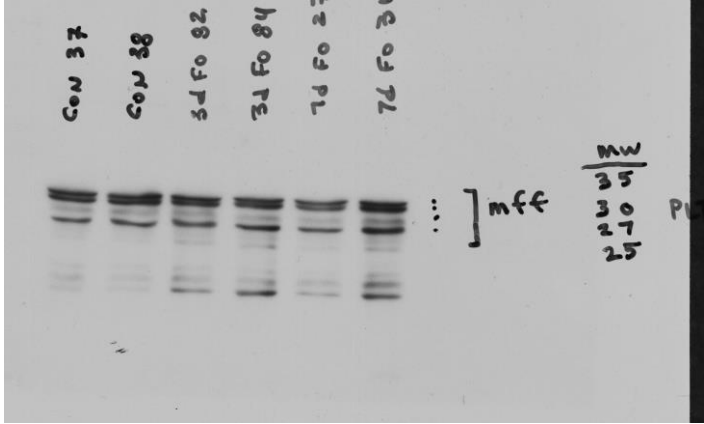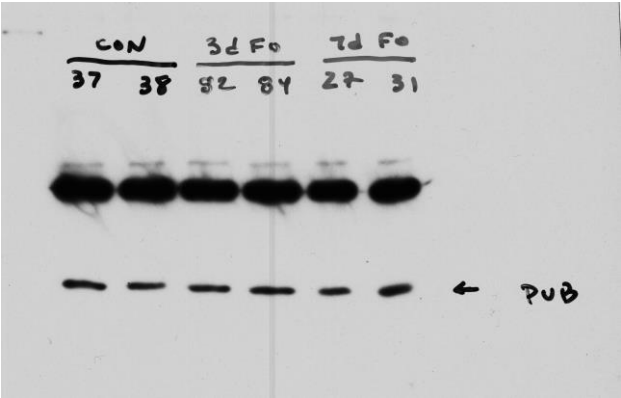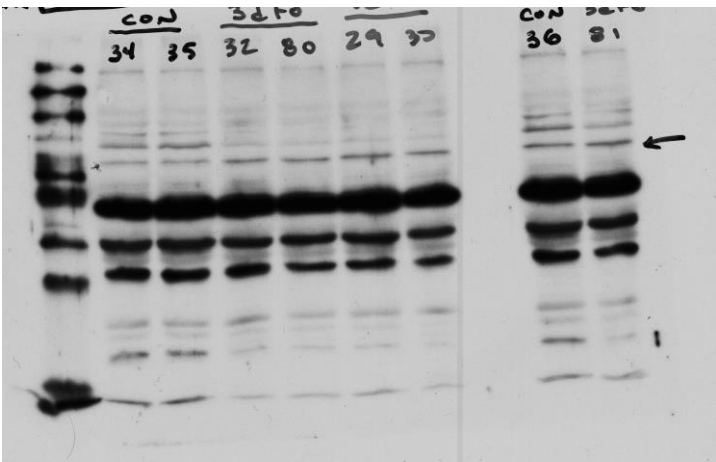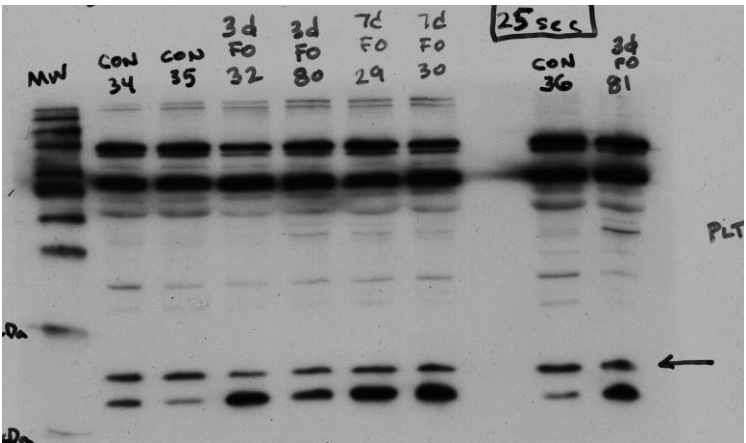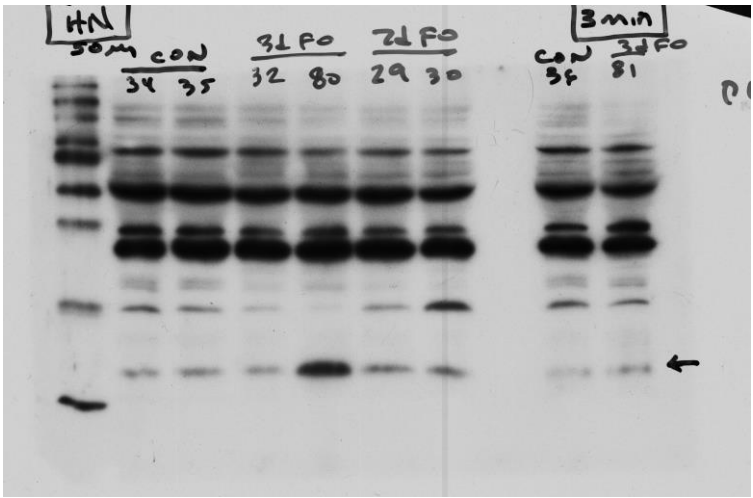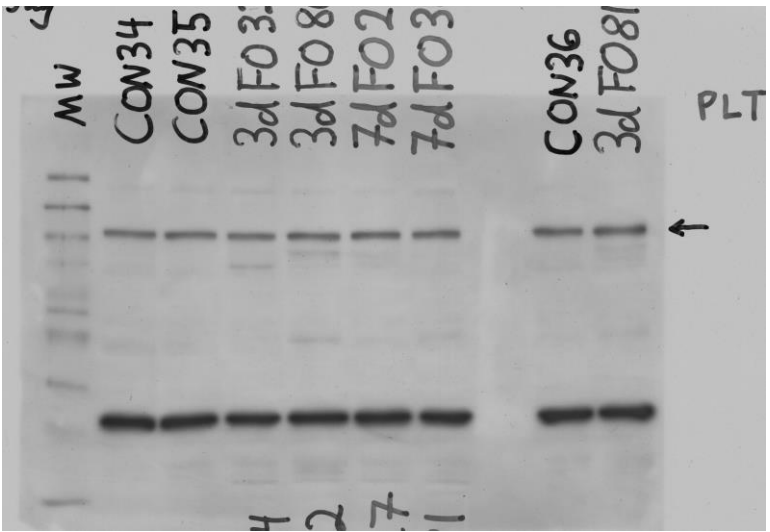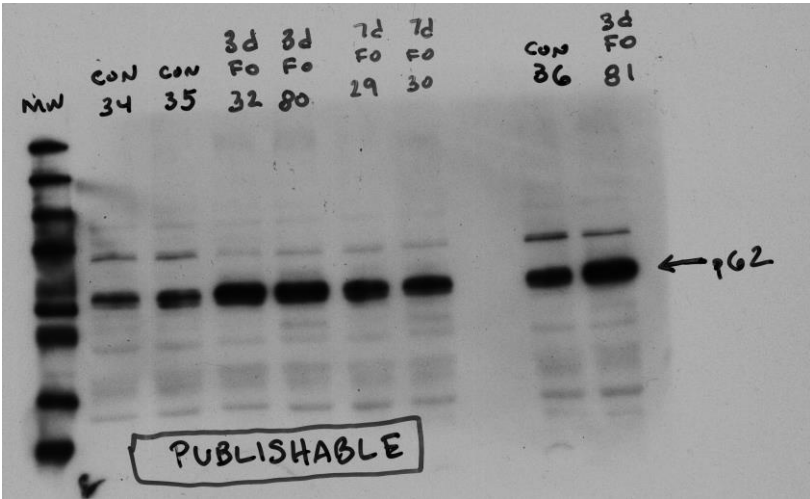

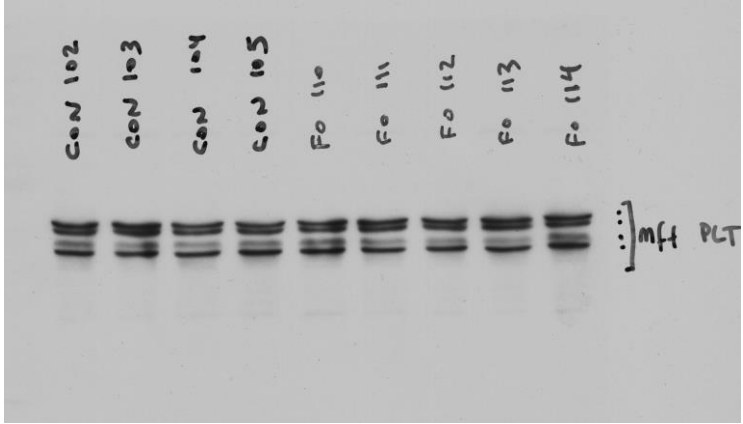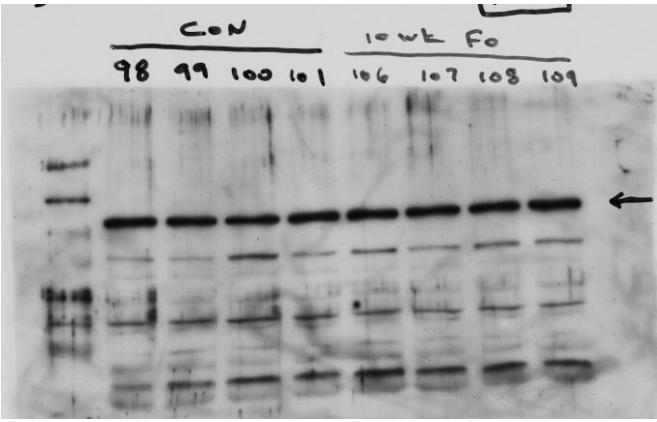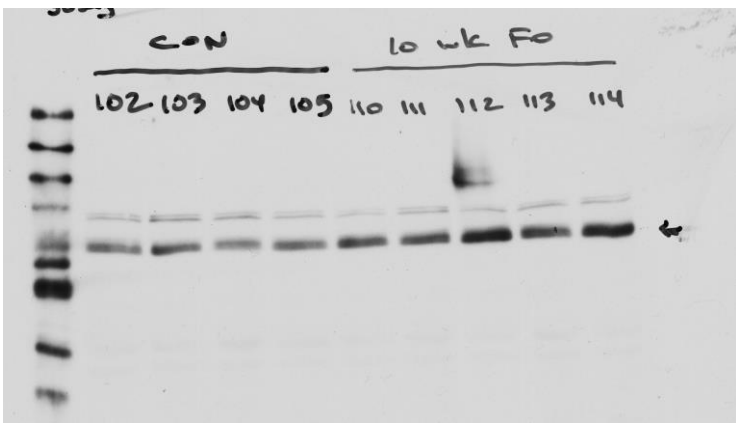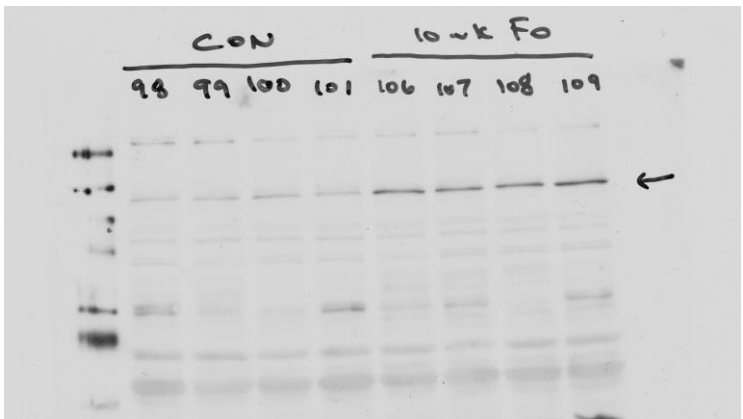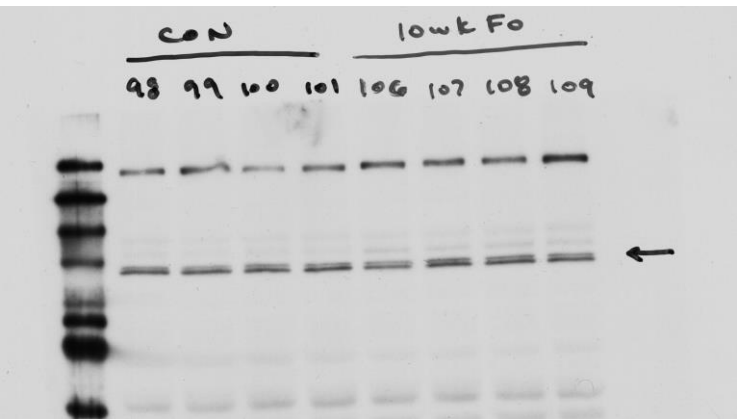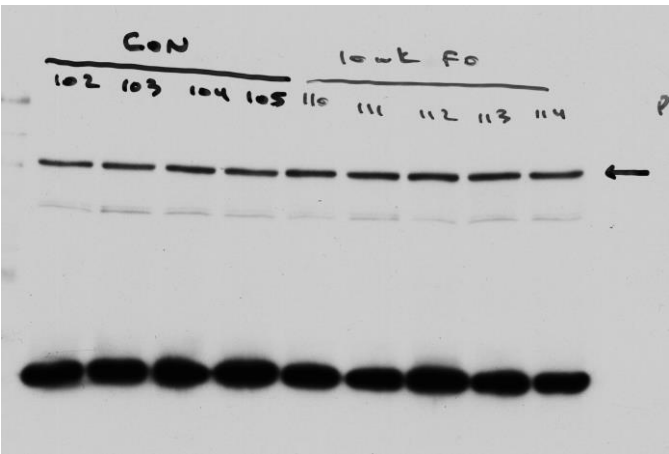

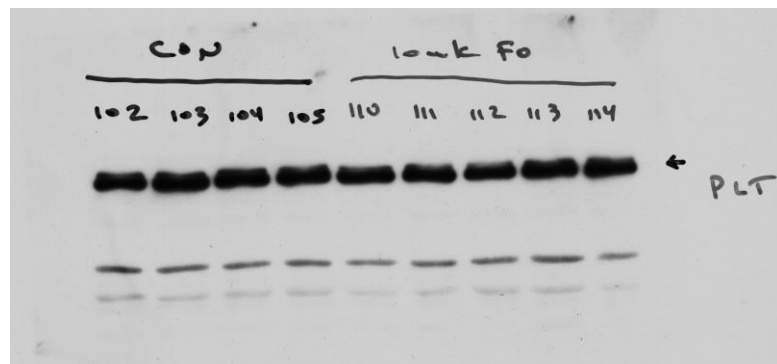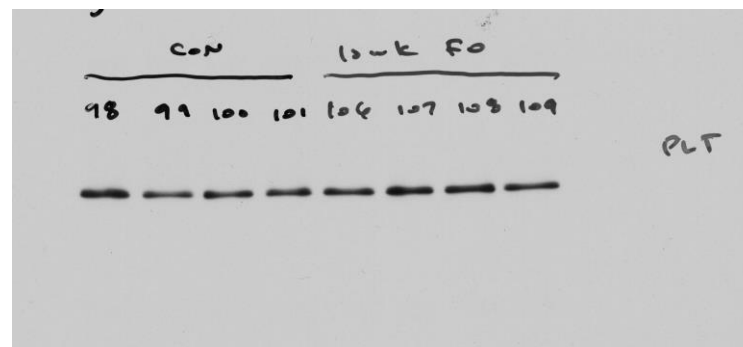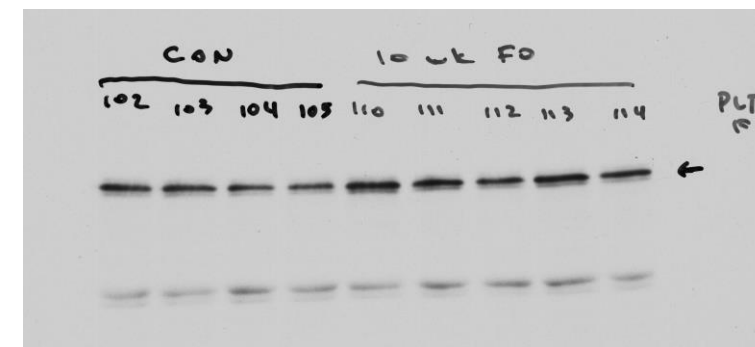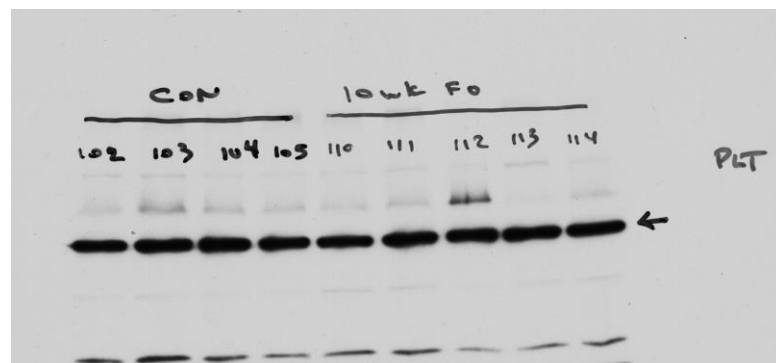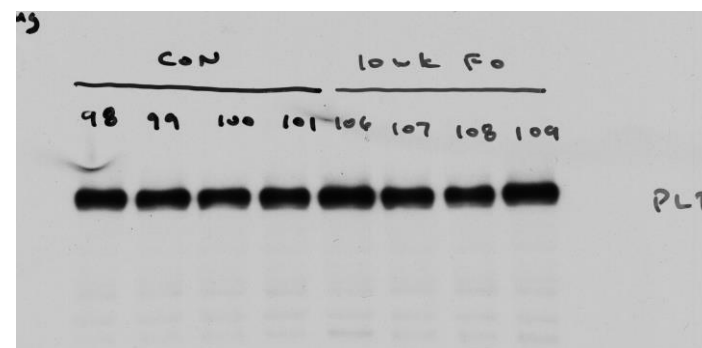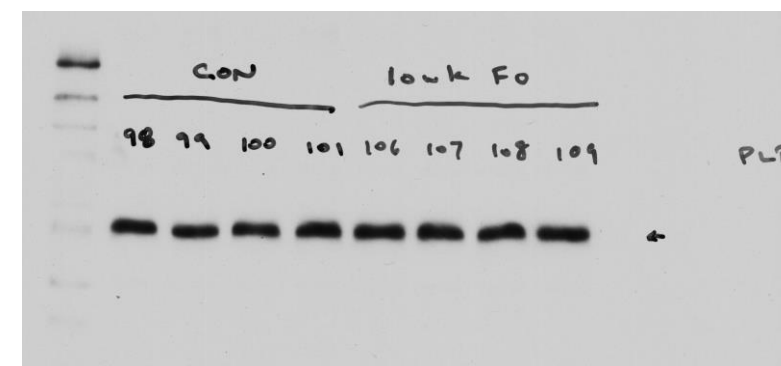

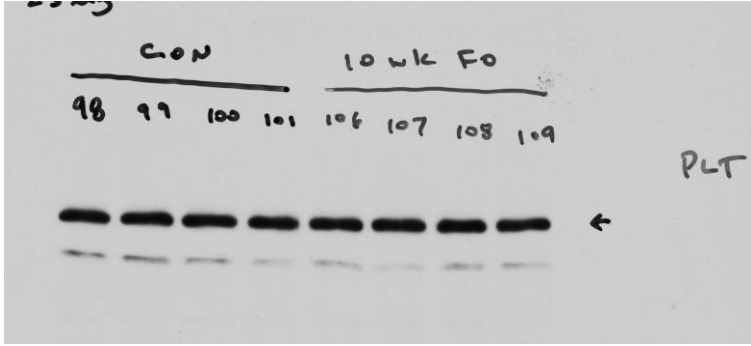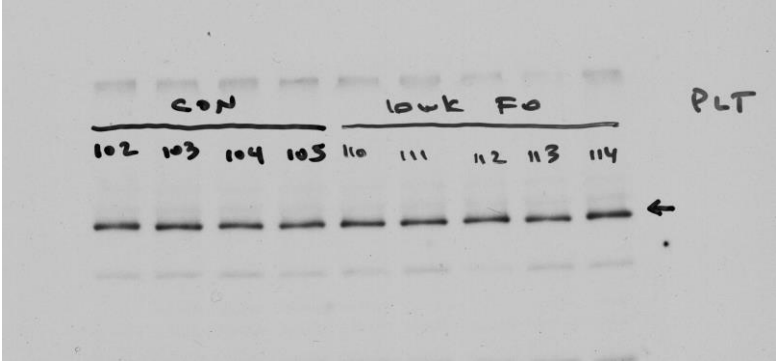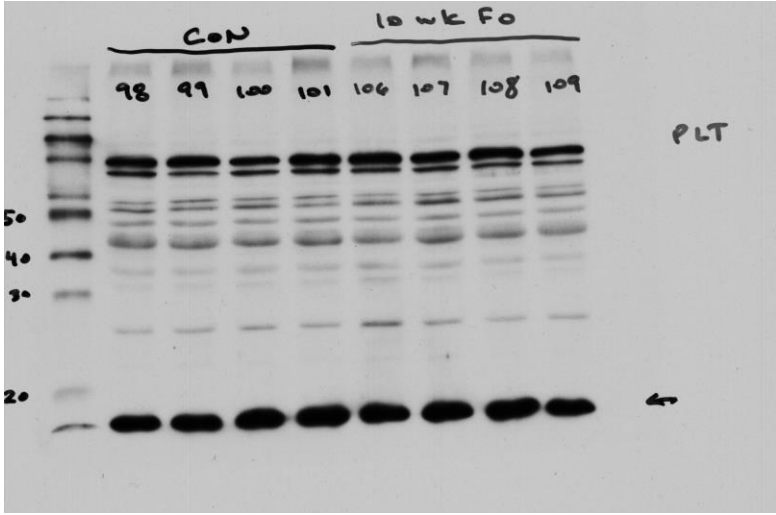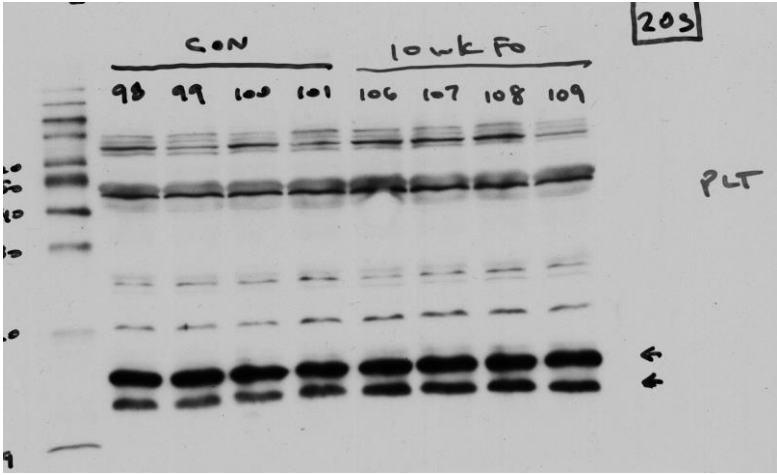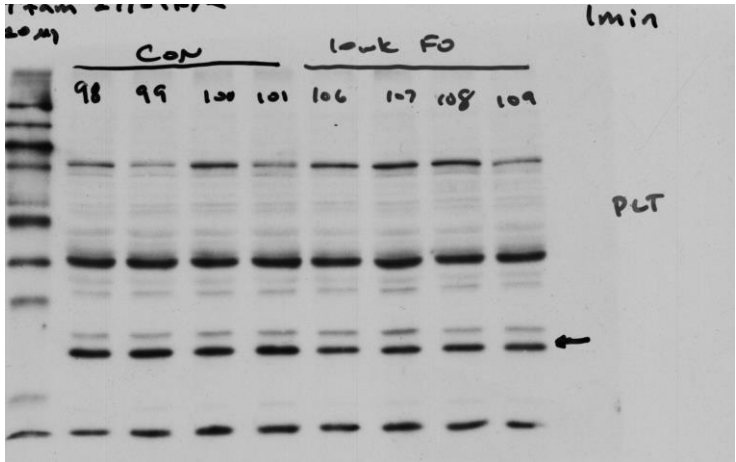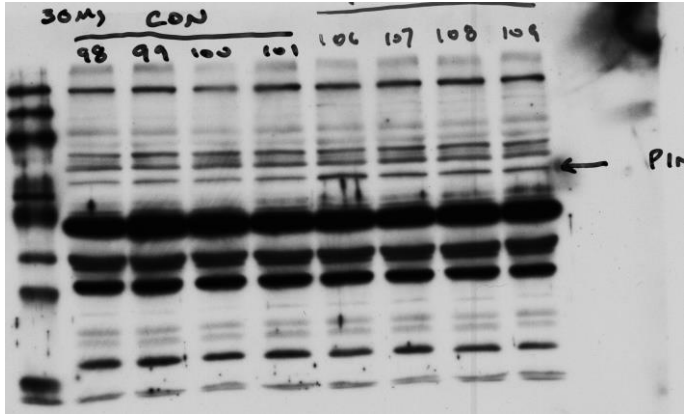

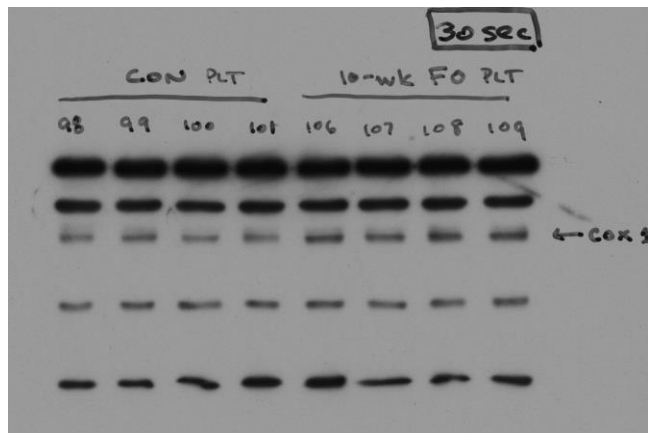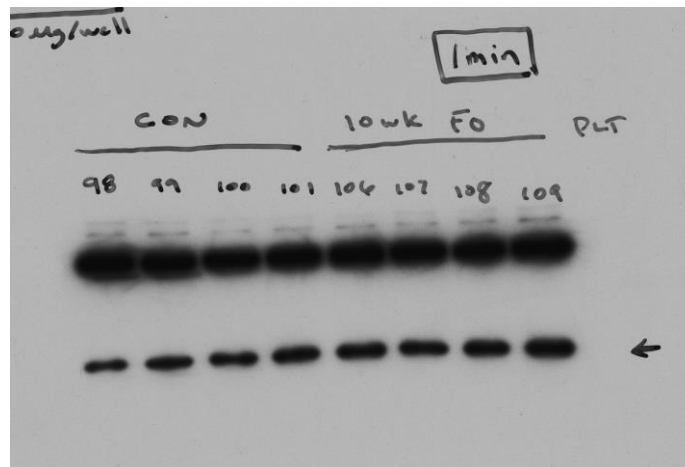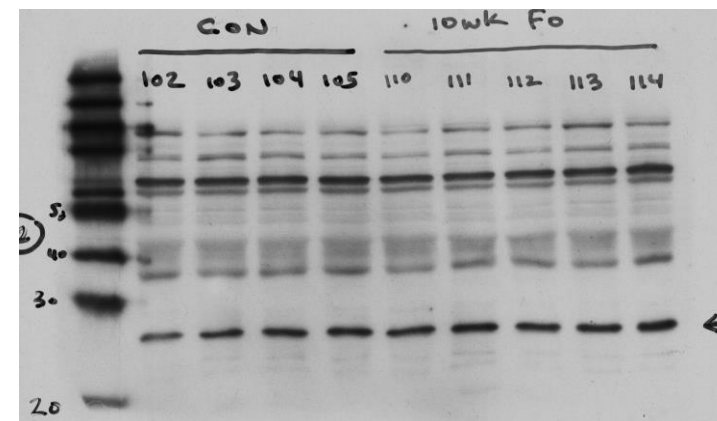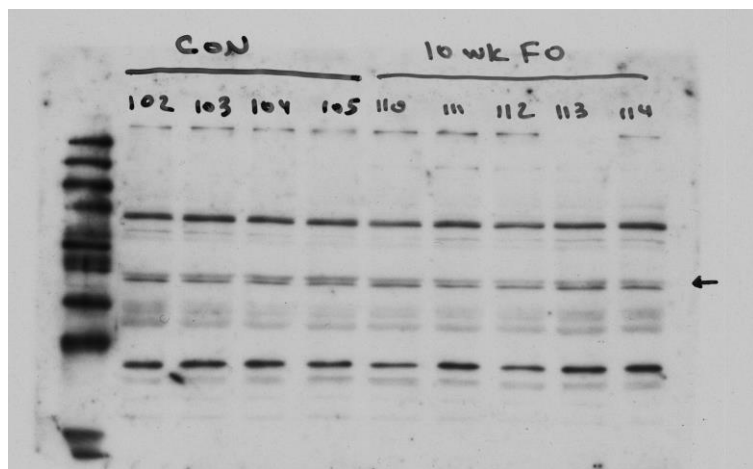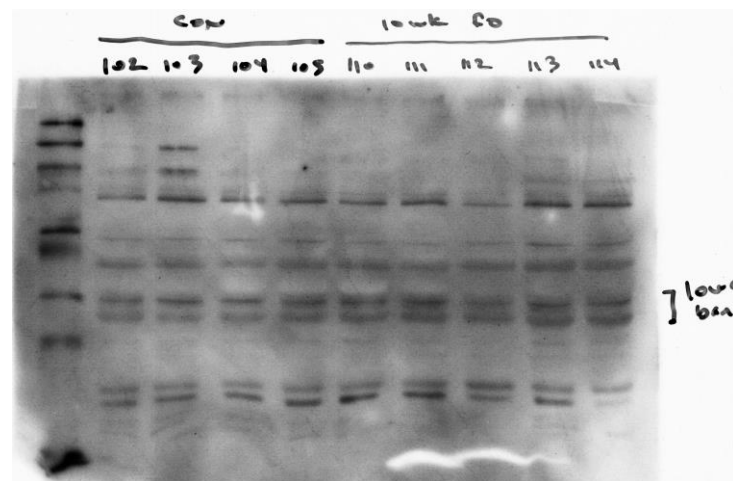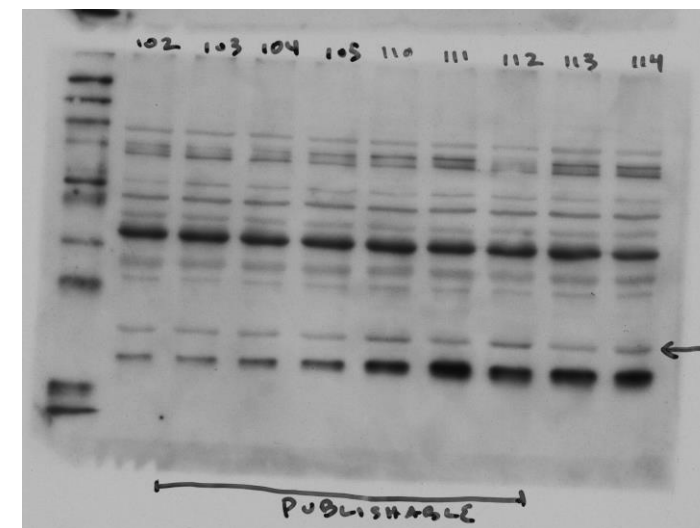

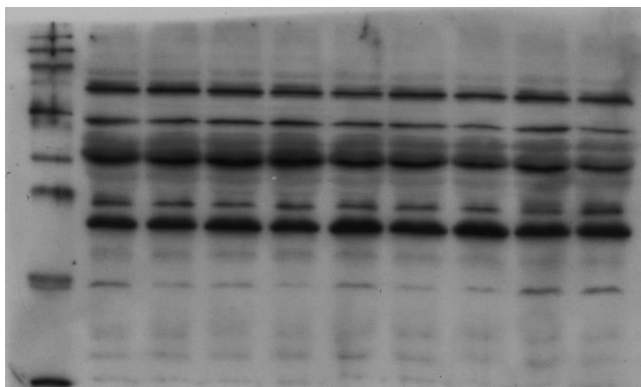

Supplement: Supplementary file 1 [file DataSheet2.PDF]
